# Biomechanical and biochemical properties of the thoracic aorta in warmblood horses, Friesian horses, and Friesians with aortic rupture

**DOI:** 10.1186/s12917-015-0597-0

**Published:** 2015-11-18

**Authors:** Veronique Saey, Nele Famaey, Marija Smoljkic, Erik Claeys, Gunther van Loon, Richard Ducatelle, Margreet Ploeg, Catherine Delesalle, Andrea Gröne, Luc Duchateau, Koen Chiers

**Affiliations:** Laboratory of Veterinary Pathology, Department of Pathology, Bacteriology and Poultry Diseases, Faculty of Veterinary Medicine, Ghent University, Merelbeke, Belgium; Biomechanics Section, Department of Mechanical Engineering, KU Leuven, Leuven, Belgium; Department of Animal Production, Ghent University, Melle, Belgium; Department of Large Animal Internal Medicine, Faculty of Veterinary Medicine, Ghent University, Merelbeke, Belgium; Department of Pathobiology, Faculty of Veterinary Medicine, Utrecht University, Utrecht, The Netherlands; Department of Equine Sciences, Faculty of Veterinary Medicine, Utrecht University, Utrecht, The Netherlands; Department of Comparative Physiology and Biometrics, Faculty of Veterinary Medicine, Ghent University, Merelbeke, Belgium

**Keywords:** Horse, Aorta, Rupture, Tensile test, Collagen, Elastin

## Abstract

**Background:**

Thoracic aortic rupture and aortopulmonary fistulation are rare conditions in horses. It mainly affects Friesian horses. Intrinsic differences in biomechanical properties of the aortic wall might predispose this breed. The biomechanical and biochemical properties of the thoracic aorta were characterized in warmblood horses, unaffected Friesian horses and Friesians with aortic rupture in an attempt to unravel the underlying pathogenesis of aortic rupture in Friesian horses. Samples of the thoracic aorta at the ligamentum arteriosum (LA), mid thoracic aorta (T1) and distal thoracic aorta (T2) were obtained from Friesian horses with aortic rupture (A), nonaffected Friesian (NA) and warmblood horses (WB). The biomechanical properties of these samples were determined using uniaxial tensile and rupture assays. The percentages of collagen and elastin (mg/mg dry weight) were quantified.

**Results:**

Data revealed no significant biomechanical nor biochemical differences among the different groups of horses. The distal thoracic aorta displayed an increased stiffness associated with a higher collagen percentage in this area and a higher load-bearing capacity compared to the more proximal segments.

**Conclusions:**

Our findings match reported findings in other animal species. Study results did not provide evidence that the predisposition of the Friesian horse breed for aortic rupture can be attributed to altered biomechanical properties of the aortic wall.

**Electronic supplementary material:**

The online version of this article (doi:10.1186/s12917-015-0597-0) contains supplementary material, which is available to authorized users.

## Background

The aorta not only serves as a conduit but also plays a major role in regulating the entire cardiovascular system thanks to its biomechanical properties. Vascular biomechanical properties play a major role in cardiovascular function. Nevertheless, information about the biomechanical and biochemical composition of the equine thoracic aorta is very sparse.

Biomechanical properties of the aortic wall are influenced by its composition and organization of its main structural components [1]. The walls of large arteries are composed primarily of elastin, collagen and vascular smooth muscle cells. Elastin supports passive wall forces at low values of wall strain. At high pressure, the resistance to stretch is mainly attributed to collagen [2]. Characterizing the biomechanical properties of soft biological materials is challenging as they are non-homogeneous and anisotropic [1]. Biomechanical properties of the aorta are usually determined by tensile testing. A method commonly applied is uniaxial tensile testing in which tissue is stretched in a controlled way from a quasi zero-stress state to rupture. During the subjection to extension, the force (or displacement) is recorded. Force-extension data can then easily be converted into stress and strain measurements [3].

Atraumatic, spontaneous aortic ruptures are uncommon and life-threatening in humans. In most cases, the aortic rupture occurs in the abdominal part and is associated with an aneurysm [4]. In non-Friesian horse breeds, aortic rupture is rare. It typically occurs at the level of the sinuses of Valsalva. In most cases it leads to acute death [5]. In Friesian horses, however, thoracic aortic ruptures are much more frequently encountered compared to other horse breeds [6]. The rupture is typically transverse near the ligamentum arteriosum. It often leads to the formation of a pseudoaneurysm and fistulation into the pulmonary artery. Survival up to several months has been reported [7].

To the best of the authors’ knowledge, tensile properties of the thoracic aorta have only been measured in hereditary equine regional dermal asthenia (HERDA) in affected and healthy Quarter Horses [8]. The purpose of the present study was to obtain reference data concerning the strength of the equine thoracic aorta and to investigate the possible role of tensile strength in aortic rupture in Friesian horses.

## Methods

### Animals and aorta preparation

*Group A consisted of 8 affected Friesian horses (4–10 years old, median age 5.4 years, 1 stallion, 3 mares and 4 geldings) that were diagnosed with aortic rupture by post mortem examination at the Faculty of Veterinary Medicine of Ghent University, Belgium. In the group of affected Friesian horses, time between death and tissue harvesting varied between 1 and 24 h.
*Group NA comprised 10 nonaffected Friesian horses (1–10 years old, median age 5.5 years, 2 geldings and 8 mares). The animals were presented for post-mortem examination at the Faculty of Veterinary Medicine of Ghent University, Belgium, for reasons unrelated to the cardiovascular system.
*Group WB consisted of 10 warmblood horses (1–10 years old, median age 5.4 years, 1 gelding and 9 mares). The horses of group 3 were presented for post-mortem examination at the Faculty of Veterinary Medicine of Ghent University, Belgium, for reasons unrelated to the cardiovascular system (*n* = 5) or were collected at the slaughterhouse (*n* = 5).

All of the patients that were admitted alive to the University hospital, were treated following the institutional guidelines. A formal ethical approval was waived by the chairperson of the ethical committee, based on Belgian and European legislation (EU directive 2010/63/EU), as all tissues were derived post mortem from the necropsy room or from a commercial abattoir.

The complete thoracic aorta, from semilunar valves to the diaphragm, was dissected and the surrounding connective/fat tissue was removed. Each thoracic aorta was cooled (4–5 °C) for maximum 8 h in isotonic physiological solution (phosphate buffered saline (PBS, pH:7.4). The time between sample harvesting and testing varied between 4 weeks and 6 months.

### Tensile and rupture test

A rectangular and a dumbbell-shaped sample were cut in axial direction at 3 different locations: adjacent to the ligamentum arteriosum (Botalli; avoiding the area of rupture and the fibrotic scar of the ligamentum) (LA), at the mid region of the thoracic aorta (T1) and at distal end of the thoracic aorta (T2).

Rectangular specimens (1 × 6 cm) were cut using a custom made cutting knife with razor blades. Dumbbell-shaped specimens (3.5 × 5.5 cm) were cut using a template and surgical scissors. Original thickness (t_0_) and width (w_0_) of each strip were measured at the zero-stress state using a digital caliper. The strips were kept frozen in PBS (−20 °C) until testing, at which point they were thawed slowly and tested at room temperature. In 3 of the 5 affected Friesian horses used for tensile testing, sampling at the level of the ligamentum arteriosum (LA) was impossible due to extensive rupture at this site. T2 samples from the 5 slaughtered warmblood horses could not be collected.

For the cyclic tensile assay, rectangular aortic samples were fixed in a set of grips and mounted on an Instron® 5567 uniaxial tensile testing bench.^1^ To avoid slipping, sandpaper was placed between the tissue and the grips. The load cell had a capacity of maximum 1kN with a resolution of 0.05N. The grip-to-grip length of each sample was manually entered based on the calculation of this length at 0 % strain by the chosen program. Both the original width and thickness, as well as the grip-to-grip length, were manually entered in the program and were thus all taken into account for calculation of the aortic properties.

The cyclic tensile test was performed at a crosshead speed of 1mm/s (10 cycles at 10 %, 10 cycles at 25 % and 10 cycles at 50 % strain). Then the samples were left to relax for 30 s at 50 % extension, followed by stretching until rupture. At each time point i, the tensile force (F_i_) and gauge length (l_i_) were recorded at a sampling rate of 10 Hz. Assuming incompressibility of the tissue, the true stress (σ_i_) and true strain (ɛ_i_) response of the tissue were derived from the force-displacement measurements as follows:

1 $$ {\sigma}_i = \frac{F_i}{A_i}, $$

2 $$ {\varepsilon}_i= log\left(\frac{l_i-{l}_0}{l_0}+1\right), $$

with ${A}_i=\frac{w_0.{t}_0.{l}_0}{l_i}$ the cross-sectional area of the sample at a certain time point i.

**Fig. 1:**
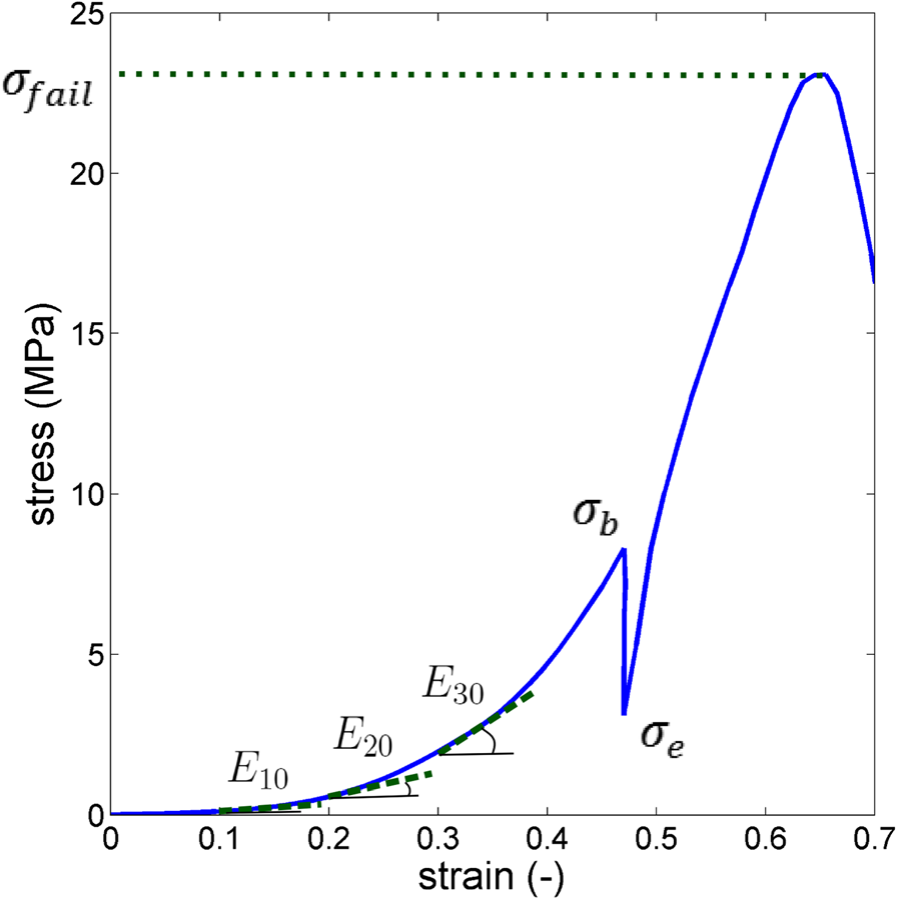
Sample stress strain curve. E_10_, E_20_ and E_30_ are the tangent stiffness moduli. The maximum engineering stress at failure is presented as σ_fail_, σ_b_ and σ^e^ are the tensile stresses at the beginning and end of the relaxation phase
- τ_r_(s): the relaxation time during the relaxation phase of the cyclic tensile test. This is a measure of the viscoelasticity of the material and is the ratio of the viscosity ŋ to the tangent elasticity *E*:
  $$ {\tau}_r=\frac{\eta }{E}=-\frac{t^{hold}}{ \log \left(\frac{\sigma_e}{\sigma_b}\right)}, $$

Data analysis was performed using Matlab® and following parameters were calculated from the stress-strain curves:

- E_10_, E_20_, E_30_ and E_40_(MPa): the tangent stiffness moduli or Young moduli at 10, 20, 30 and 40 % strain, which are defined as the slopes of the stress-strain curve at the respective strain level. As the aorta is a nonlinear material, a single value of elastic Young’s modulus would not represent the stiffness of the material. A typical curve obtained when determing the tangent stiffness is depicted in Fig. 1.

With t^hold^ =30s, i.e., the time at which the crosshead remained in place, and σ_b_ and σ_e_ the tensile stress at the beginning and at the end of the relaxation phase, respectively. Note that a higher relaxation time corresponds to a lower viscoelasticity.

For the rupture test, dumbbell shaped aortic samples were mounted on the same tensile machine. At the same crosshead speed of 1mm/s, the sample was elongated until rupture. Again, tensile force and gauge length were recorded. Two specimen modes of failure were used as data exclusion criteria: edge slipping out of the grips or when failure did not occur at the necked region of the dumbbell-shaped specimens. From these tests, the following parameter was extracted using Matlab®:

- ${\sigma}_{fail}=\frac{F_{max}}{A_0}\left(\mathrm{M}\mathrm{P}\mathrm{a}\right)$: the engineering stress value at which failure occurred, a measure of the strength of the material. In the equation, F_max_ is the maximum force recorded during the tensile experiment and A_0_ = w_0_.t_0_ s the initial cross-sectional area of the sample at the necked region.

For a more detailed explanation of the variables defined here, see Dill 2006 [9], chapters 2.5 and 5.1.

### Collagen and elastin quantification

Quadratic samples of the aorta (5 × 5 cm) were cut using surgical scissors at the three levels as mentioned above and stored frozen (−20 °C).

For collagen analysis, one gram of frozen aortic tissue was oven dried for 2 h at 100 °C. Dry weight was determined. The collagen concentration was then quantified using the ISO/DIS 3496.2 method [10] and was expressed as percentage of dry weight.

For elastin analysis, one gram of frozen aortic tissue was first pulverized in liquid nitrogen using a B-Braun Biotech International mikro-dismembrator.^2^ Elastin was then prepared by the hot alkali method [11], a modification of the original method of Lansing et al. [12]. This method is based on the degradation of the peptide bonds in elastin [13]. The remaining pellet was dried by lyophilisation for 24 h to yield elastin weight fractions. Elastin concentration was expressed as percentage of dry weight. The purity of the elastin was confirmed by histology and immunohistochemistry. From each group of horses, an elastin pellet was embedded in paraffin wax, and cut into 4-μm-thick sections. Sections were stained with hematoxylin and eosin which showed fragmented eosinophilic fibrillary material without nuclei (compatible with elastin fibers). All fibers present on the slides stained positive with a monoclonal anti-elastin Leica Bio-systems antibody BA-4.^3^ A standard Envision avidin-biotin complex method^4^ with diaminobenzidine as chromogen was used for visualization.

Collagen and elastin quantification was not performed in the 5 slaughtered warmblood horses.

### Statistical analysis

Statistical analyses were based on a mixed model with horse nested in group as random effect and location (LA/T1/T2), group of horse (A/NA/WB) and their interaction as fixed effects. For the analysis of the modulus, the strain level was added to the model as a fixed effects factor. The normality assumption was tested by the Shapiro Wilk test and was not rejected. Overall F-tests and pairwise comparisons were performed. A Bonferroni correction was applied for the pairwise comparisons between horse groups with comparisonwise significance level set at 0.0166 (=0.050/3).

## Results

### Tensile and tearing test

**Fig. 2:**
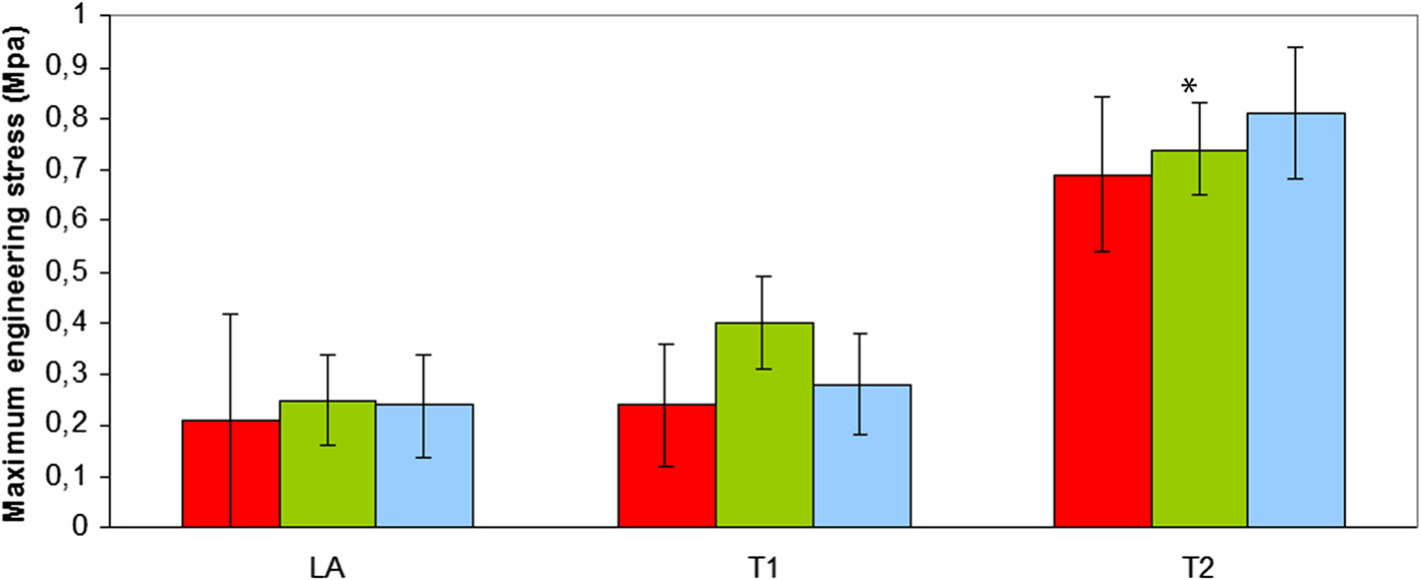
Maximum engineering stress at failure of the equine thoracic aorta at 3 sample locations (LA-T1-T2) in 3 groups of horses (red: Friesian horses with aortic rupture; green: nonaffected Friesian horses; blue: nonaffected warmblood horses) (*: *p* < 0.001)

**Fig. 3:**
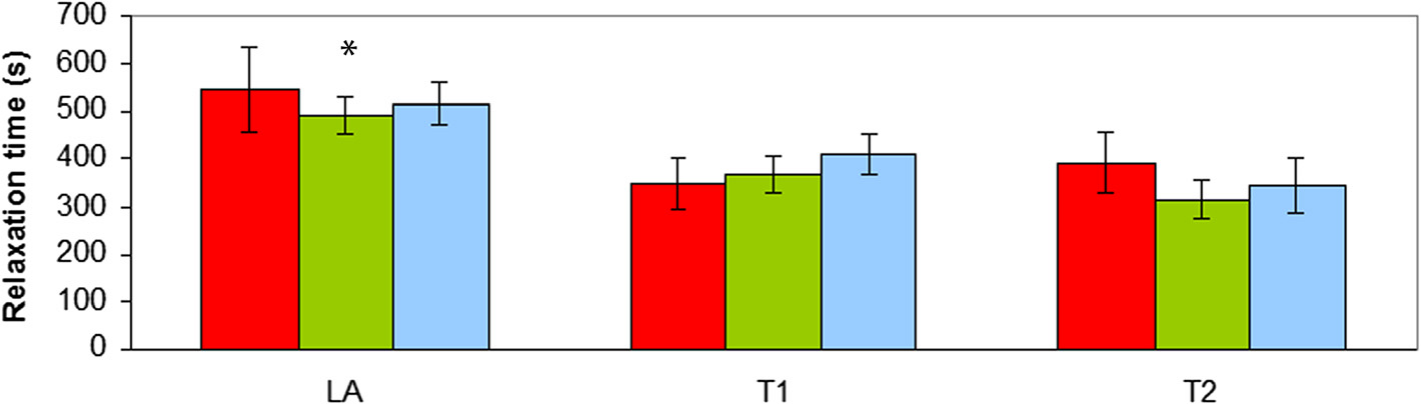
Relaxation time, determined during cyclic testing, of the equine thoracic aorta at 3 sample locations (LA-T1-T2) in 3 groups of horses (red: Friesian horses with aortic rupture; green: nonaffected Friesian horses; blue: nonaffected warmblood horses) (*: *p* < 0.002)

Additional file 1 no significant differences between the three groups of horses were observed for σ_fail_, τ_r_ and E_i._ and also no significant interactions between group of horse and location were found (*p* > 0.05) There was however a significant overall effect (over the horse groups) of the location on these parameters. The σ_fail_ (MPa), the tissue strength, was significantly (*p* < 0.001) higher at location T2 compared to LA and T1 (B: 0.22 ± 0.06; T1: 0.31 ± 0.06; T2: 0.73 ± 0.07) (Fig. 2). The tangent moduli E_i._ of the aorta at location LA and T1 was lower (*p* < 0.001) compared to location T2 (LA: 0.19 ± 0.05; T1: 0.29 ± 0.05; T2: 0.70 ± 0.05). ^τ^The relaxation time (s) was higher at location LA compared to T1 and T2 (LA: 508 ± 29; T1: 381 ± 26; T2: 343 ± 30) (*p* < 0.002) (Fig. 3).

### Collagen and elastin concentration

**Fig. 4:**
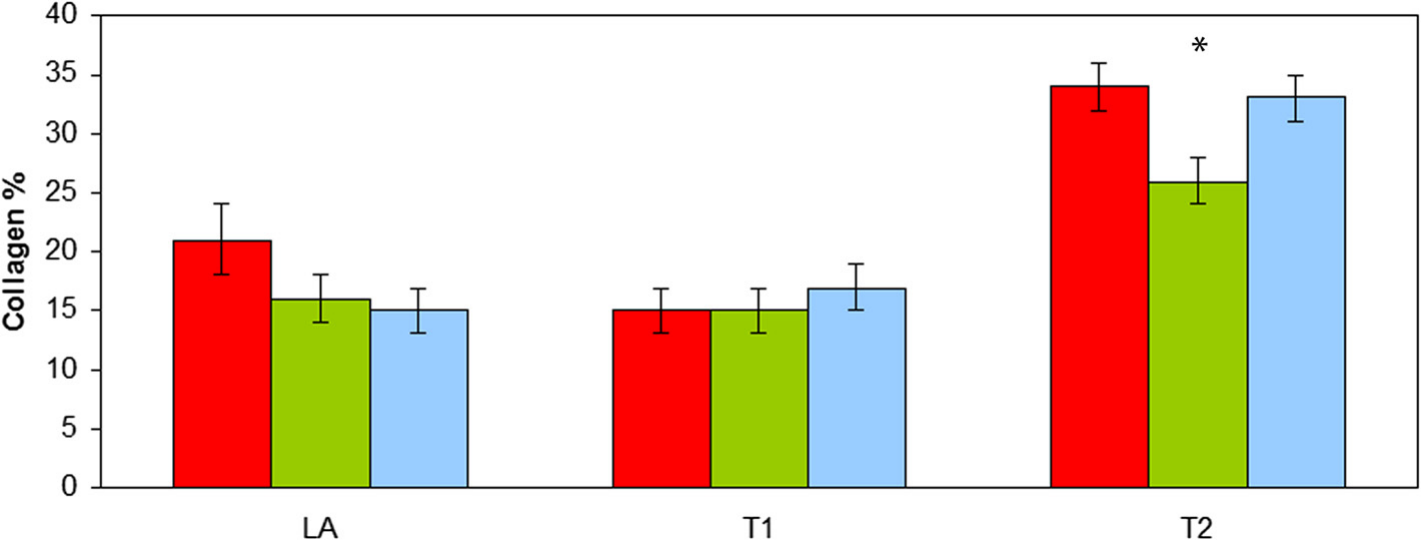
Collagen percentage of the equine thoracic aorta at 3 sample locations (LA-T1-T2) in 3 groups of horses (red: Friesian horses with aortic rupture; green: nonaffected Friesian horses; blue: nonaffected warmblood horses) (*: *p* < 0.001)

**Fig. 5:**
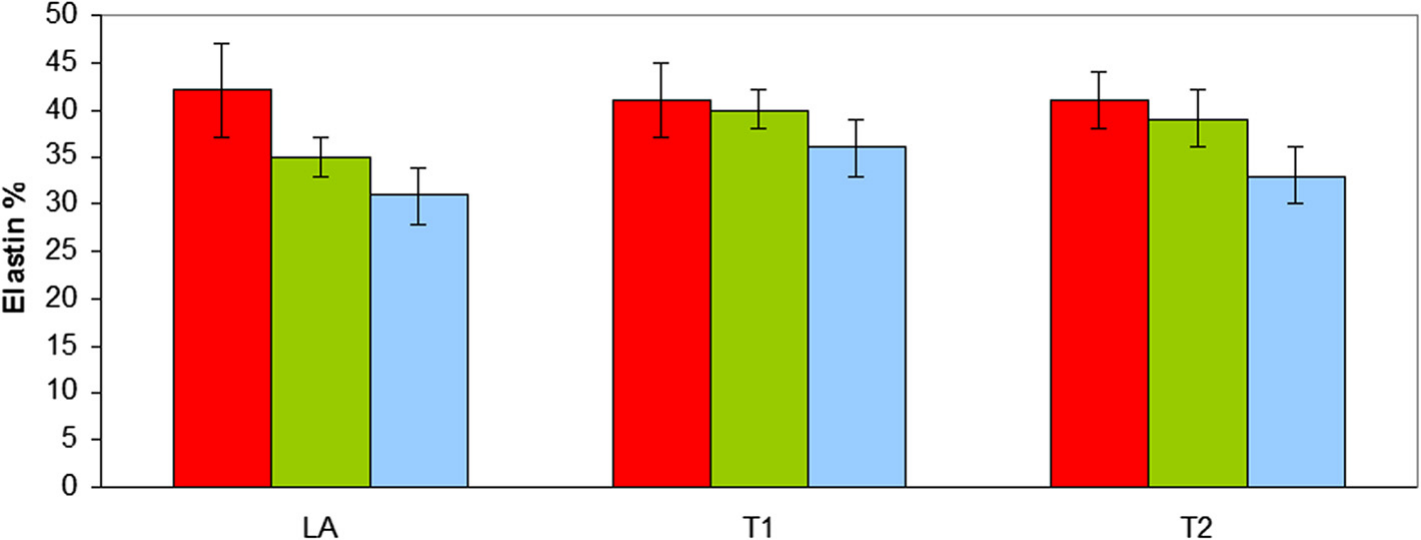
Elastin percentage of the equine thoracic aorta at 3 sample locations (LA-T1-T2) in 3 groups of horses (red: Friesian horses with aortic rupture; green: nonaffected Friesian horses; blue: nonaffected warmblood horses)

No significant effect of horse group or interaction between horse group and location was found for the collagen and elastin concentration (*p* > 0.05). The collagen concentration was significantly higher (*p* < 0.001) at T2 compared to LA and T1 (LA: 17 % ± 1; T1: 16 % ± 1; T2: 31 % ± 1). The aorta of the affected Friesians at the rupture site (LA) tended to contain a numerically higher collagen percentage compared to the non-affected Friesian and warmblood horses, however this difference was not statistically significant (*p* > 0.05) (Fig. 4). The elastin percentage showed no significant regional differences (*p* > 0.05) (Fig. 5).

## Discussion

The present study demonstrates that the biomechanical and biochemical characteristics of the equine aortic wall show regional differences. The distal site of the thoracic aorta (T2) could withstand a higher maximum stress and was stiffer compared to the more proximal sites (LA and T1). The aorta at the ligamentum arteriosum (LA) was less visco-elastic compared to T1 and T2. These regional differences are in accordance with the majority of reports in the literature confirming the regional variation of the biomechanical properties of the aorta in humans and several animal species [14–16]. In canine aortas, the force needed to rupture longitudinal strips is minimal in the ascending aorta and in general increases with distance from the semilunar valves [14]. In our study, the equine distal thoracic aorta (T2) was also able to withstand higher maximum stresses before rupture compared to the more proximal thoracic aorta (LA and T1).

In the study by Bowser et al. [8], samples were collected from the thoracic aorta of Quarter Horses (6 HERDA affected - 6 control) with a similar median age as in our study. The tensile strength of the thoracic aorta in the HERDA study of the control Quarter Horses (0.45 MPa) was comparable to the average failure stress σ_fail_ of our samples (0.42 MPa). To compare the tangent moduli to the elastic modulus, the method used in the present study is preferred for two reasons. First, we report the tangent modulus related to the true stress-strain curve, whereas Bowser et al. [8] reported the slope of the engineering stress-strain curve. The latter is based on the initial cross-sectional area of the sample and underestimates the stiffness for large deformations. Secondly, we report a number of tangent moduli along the nonlinear stress-strain curve, thereby accounting for the material nonlinearity, whereas Bowser et al. [8] reported one linearized (or averaged) value. Other explanations for differences in stiffness can be attributed to breed differences and the difference in location of sampling.

In Friesian horses, an underlying genetic defect of the connective tissue in the aortic media has been suggested based on the morphological characteristics of the lesions [17]. The higher percentage of collagen near the ruptured site of the affected Friesian horses, although not significant, can be explained by the medial fibrosis seen at the level of the aortic rupture histologically. This was suggested to be due to chronic injury or abnormal hemodynamic changes [17]. As the aortic rupture in Friesian horses typically occurs near the scar of the ligamentum arteriosum [18], samples at this site (LA) were included and biomechanical properties were determined. Unfortunately, as mentioned above, due to the small number of samples of affected Friesians and because the aortic rupture is relatively large, it is likely that the exact predisposed site in susceptible animals was not included. Friesians were not different from warmblood horses in their biomechanical properties. Based on our results, it is unlikely that the aortic rupture is associated with a generalized aortic wall condition in Friesians.

The average sum of the collagen and elastin content in all horses ranged from 55 % at location LA to 56 % at T1 and 67 % at T2. This is in accordance with the reported content of about 60 % of collagen plus elastin in the aortic media, regardless of species [19].

The distal thoracic aorta (T2) was stiffer compared to the proximal thoracic aorta (LA and T1) and contained a higher percentage of collagen. It is generally accepted that, due to the predominance of collagen fibers, the distal aorta becomes stiffer [20]. In contrast, elastin content is reported to be equally distributed along the thoracic aorta [21], as was also found in our study. Zou and Zhang [21] also suggested that the biomechanical properties of elastin can vary along the thoracic aorta. Indeed, solely the arteries’ collagen and elastin content is not sufficient to explain the differences in elastic moduli at different sites, but depends on their in vivo biomechanical properties and architectural arrangement [22].

In human Marfan syndrome, a hereditary disorder of the fibrillar metabolism, cystic medial necrosis is a typical finding and associated loss of elastin has been mentioned [23]. Perejda et al. [24] were able to demonstrate a decreased tensile strength of the aorta in affected patients. In the histological study of aortic lesions in Friesians with aortic rupture, cystic medial necrosis was reported [17]. In the affected Friesians in this study, there was no decrease in elastin concentration and mechanical properties were not altered near the area of the rupture. In bovine Marfan syndrome however, cystic medial necrosis is absent and elastin concentrations are reported to be normal [25]. True aneurysms are not observed in aortic rupture in Friesian horses [7] and there are no indications pointing towards a Marfan-like syndrome in these horses.

Our method of testing shows some limitations as only uniaxial testing on aortic strips was performed. Excised aortic strips retract leading to thickening of the wall and reorientation of intramural structures [26]. As this test method ignores the multiaxial loading state under physiological conditions, it gives thus no information on the anisotropy and the relation between pressure and radius in the intact vessel [27]. All measurements were performed in the axial direction, while under physiological conditions, the aorta is subjected to cyclic strains in both circumferential and longitudinal direction. In Friesian horses, aortic rupture typically occurs transversely [7], indicating a possible weakening of the aorta and thus a decreased tensile strength. Therefore, axial testing of the aorta in longitudinal direction was preferred in this study as we wanted to compare the tensile strength of the aorta between horse breeds. Due to practical reasons, it was impossible to create a standardized time between tissue harvesting and testing. However, we strongly believe that this did not affect our results since we assured similar time periods at similar degrees of samples of the three different groups. Moreover, performing the measurements in batches increased the repeatability of the tests.

Note that the variables discussed here pertain to the material properties of the artery and hence make abstraction of the arterial geometry. This is a conscious choice, as it allows a fair comparison of the intrinsic material without having to take into account the complex geometry and loading state in each animal.

## Conclusions

The equine thoracic aorta is stiffer more distally. This can be explained at least partially by a higher collagen content. No significant biomechanical and compositional differences were found between warmblood horses, Friesian horses with aortic rupture and nonaffected Friesians. Our results suggest the existence of a local, hereditary defect, rather than a generalized aortic wall condition, predisposing Friesian horses for aortic rupture. At this point, one cannot exclude a difference in organization of collagen and elastin being responsible for the rupture, however this would most probably reflect on the general biomechanical properties.

## Additional file

Additional file 1:
**Summary of the biomechanical and biochemical properties of the equine thoracic aorta in the 3 groups of horses.** (XLSX 26 kb)

## Abbreviations

LA: Thoracic aorta at the ligamentum arteriosum
T1: Mid thoracic aorta
T2: Distal end of the thoracic aorta
A: Friesian horses with aortic rupture
NA: Nonaffected Friesian horses
WB: Nonaffected warmblood horses
HERDA: Hereditary equine regional dermal asthenia
PBS: Phosphate buffered saline

## Footnotes

1 Instron 5567, Norwood, USA.

2 B-Braun Biotech International, Melsungen, Germany.

3 Leica Bio-systems, Diegem, Belgium.

4 Envision, Dako, Glostrup, Denmark.
